# Biomechanical and clinical outcomes of 3D-printed versus modular hemipelvic prostheses for limb-salvage reconstruction following periacetabular tumor resection: a mid-term retrospective cohort study

**DOI:** 10.1186/s13018-024-04697-w

**Published:** 2024-04-23

**Authors:** Xin Hu, Yang Wen, Minxun Lu, Yi Luo, Yong Zhou, Xiao Yang, Chongqi Tu, Li Min

**Affiliations:** 1 Department of Orthopedic Surgery and Orthopedic Research Institute, West China Hospital, Sichuan University, No. 37 Guo Xue Xang, Chengdu, 610041 Sichuan People’s Republic of China; 2 Model Worker and Craftsman Talent Innovation Workshop of Sichuan Province, No. 37 Guoxue Road, Chengdu, 610041 Sichuan People’s Republic of China; 3 National Engineering Research Center for Biomaterials, Sichuan University, Chengdu, 610064 Sichuan People’s Republic of China; 4 Provincial Engineering Research Center for Biomaterials Genome of Sichuan, Sichuan University, Chengdu, 610064 People’s Republic of China; 5 Department of Orthopedics, Zigong Fourth People’s Hospital, Zigong, 643000 People’s Republic of China

**Keywords:** Hemipelvectomy, 3D-printed, Sarcoma, Surgical reconstruction, Modular, Oncology

## Abstract

**Background:**

Debates persist over optimal pelvic girdle reconstruction after acetabular tumor resection, with surgeons grappling between modular and 3D-printed hemipelvic endoprostheses. We hypothesize superior outcomes with 3D-printed versions, yet scarce comparative research exists. This study fills the gap, examining biomechanics and clinical results retrospectively.

**Methods:**

From February 2017 to June 2021, we retrospectively assessed 32 patients undergoing en bloc resection for malignant periacetabular tumors at a single institution. Primary outcome: limb function. Secondary outcomes: implant precision, hip joint rotation center restoration, prosthesis-bone osteointegration, and complications. Biomechanical characteristics were evaluated through finite element analysis on pelvic defect models.

**Results:**

In the 3D-printed group, stress distribution mirrored a normal pelvis, contrasting the modular group with elevated overall stress, unstable transitions, and higher stress peaks. The 3D-printed group exhibited superior functional scores (MSTS: 24.3 ± 1.8 vs. 21.8 ± 2.0, *p* < 0.05; HHS: 79.8 ± 5.2 vs. 75.3 ± 3.5, *p* < 0.05). Prosthetic-bone interface osteointegration, measured by T-SMART, favored 3D-printed prostheses, but surgery time (426.2 ± 67.0 vs. 301.7 ± 48.6 min, *p* < 0.05) and blood loss (2121.1 ± 686.8 vs. 1600.0 ± 505.0 ml, *p* < 0.05) were higher.

**Conclusions:**

The 3D-printed hemipelvic endoprosthesis offers precise pelvic ring defect matching, superior stress transmission, and function compared to modular endoprostheses. However, complexity, fabrication expertise, and challenging surgical implantation result in prolonged operation times and increased blood loss. A nuanced consideration of functional outcomes, complexity, and patient conditions is crucial for informed treatment decisions.

**Level of evidence:**

Level III, therapeutic study (Retrospective comparative study).

**Supplementary Information:**

The online version contains supplementary material available at 10.1186/s13018-024-04697-w.

## Introduction

Primary malignant bone tumors involving the pelvic girdle constitute around 10–15% of cases [11, 20]. Currently, limb-salvage surgeries are the primary surgical treatment for malignant pelvic bone tumors, owing to advancements in adjuvant chemotherapy, imaging, and surgical techniques [6, 8]. Surgical reconstruction options following tumor resection include arthrodesis [29], hip transposition [41], allograft/autograft reconstruction [2, 4, 34], and endoprosthetic reconstruction [1, 5, 11, 19, 20, 26, 27]. Among them, endoprosthetic reconstructions are preferred for their stability, aesthetics, early mobility, and the absence of risks of bone grafts-related complications.

In clinical practice, various hemipelvic endoprostheses are utilized, including saddle prostheses, ice-cream cone prostheses, modular prostheses, and 3D-printed hemipelvic prostheses [5, 7, 11, 18, 27]. However, saddle and ice-cream cone prostheses demand a significant volume of retained ilium for fixation, limiting their clinical application [30]. In contrast, modular hemipelvic endoprostheses offer the advantage of being assembled flexibly during pelvic tumor resection, even in cases involving extensive iliac bone resection [12, 21]. The smaller dimensions of these endoprostheses facilitate generous soft tissue coverage during surgical procedures, effectively minimizing residual dead space and ensuring robust muscle reconstruction. These advantages not only enhance the efficacy of hip joint function rehabilitation but also hold promise, in theory, for reducing the incidence of deep postoperative infections in patients [11, 22]. However, modular endoprostheses exhibit inadequate interface matching and their fixation method does not conform to optimal mechanical transmission [10]. Furthermore, the absence of osteointegration at the interface results in mechanical failures during later stages. [1, 27, 39]. Despite the incorporation of porous structures on the surface of modular prostheses by certain scholars, the fundamental issue of inadequate stress transmission persists [9, 23]. In recent years, 3D-printed hemipelvic endoprostheses have gained increasing attention [15]. The core advantage of 3D printing technology resides in its aptitude for precise customization. These innovative prostheses can be tailored to conform to any irregular pelvic bone deficiency. Moreover, the incorporation of a porous structure on the prosthesis surface promotes osteointegration [38]. As such, this technology has the potential to address the challenges of prosthesis integration with native bone and ensure its enduring stability over time. Over the recent decades, 3D-printed hemipelvic endoprostheses emerge as a promising alternative reconstruction option for patients with malignant tumors around the acetabulum [24, 37, 38, 40, 42].

Nowadays, pelvic girdle reconstruction after acetabular tumor resection lacks a consensus on the optimal approach. Surgeons face a dilemma between modular endoprostheses and emerging 3D printing technology, each with its own pros and cons. Regarding these two essential pelvic ring reconstruction methods, it appears that 3D-printed hemipelvic endoprostheses, when it comes to anatomical pelvic ring reconstruction, mimic stress transmission patterns akin to those seen in native human pelvises. However, no studies have yet been reported to ascertain whether they exhibit superior biomechanical performance compared to non-anatomical reconstruction using modular endoprostheses or if this leads to improved mid-term clinical outcomes. To the best of our knowledge, prior research on these two critical reconstruction methods has been lacking in controlled comparative studies or relevant biomechanical analysis. Herein two surgical approaches for pelvic girdle reconstruction after periacetabular tumor resection in 32 patients are compared both clinically and biomechanically in this study aimed at the identification of a better operative strategy.

## Methods

### Clinical study and patients

This single-center retrospective study was performed in accordance with the 1964 Helsinki Declaration and was authorized by the Ethics Committee of our hospital. Written informed consent was obtained from adult participants or parents of minors (below 16 years of age). The work has been reported in line with the STROCSS criteria [25].

We retrospectively analyzed the results of patients who underwent either 3D-printed or modular endoprosthestic reconstruction for the treatment of pelvic bone tumor between February 2017 and June 2021. Given the absence of significant differences in the indications for modular endoprosthesis and 3D-printed endoprosthetic reconstruction, both approaches are viable for reconstruction within the same patient. The decision on which reconstruction method to employ hinges on detailed communication with the patient, taking into account differences in postoperative reconstruction, prosthetic costs, and the time required for design and production. Ultimately, the choice is made based on the patient's preferences. In this retrospective analysis, we included patients with comparable numbers, tumor locations, and tumor sizes who participated in the study. Thus, the inclusion criteria were: (1) Pathological confirmation of a primary or metastatic pelvic malignant tumor. (2) Absence of contraindications for en bloc resection. (3) Life expectancy exceeding 6 months. (4) Comparable planned resection margin to that achieved in hemipelvectomy. (5) Expected preservation of reasonable function post-resection. (6) Utilization of 3D-printed custom-made hemipelvic endoprostheses or modular hemipelvic prostheses for reconstruction. (7) Availability of comprehensive follow-up data. The exclusion criteria were: (1) Inability to achieve a satisfactory surgical margin while pursuing limb-salvage procedures; (2) Incapacity to preserve a functional limb due to tumor involvement of the sacral or sciatic nerve; (3) Patients presenting with unresectable and/or extensively metastatic disease; (4) Active infection in the proximity of the prosthesis implantation site; (5) Allergy to metal implants; (6) Severe reduction in muscular strength of the affected limb or significant impairment of other joints impacting functional assessment; (7) Profound osteoporosis.

After careful assessment of eligibility criteria and obtaining informed consent from all participants, a total of 32 patients were included in this study. Patients with similar preoperative tumor locations (Type I + II, Type II + III, Type I + II + III) and comparable tumor sizes but differing in the surgical reconstruction approach were divided into two groups: the modular group and the 3D-printed group for a comparative study. Prior to their surgical procedures, all patients underwent a thorough pathological examination and were staged according to the Enneking classification system for tumor categorization. Furthermore, a comprehensive set of evaluations was conducted, encompassing physical examinations, biochemical analyses, and a range of imaging techniques, such as X-ray, 3D-CT, MRI, and SPECT. Thin-layer chest CT scans were specifically carried out to detect any potential lung metastases. To gauge pain levels, Visual Analog Scale (VAS) scores were documented, and the functional outcomes were assessed using the MSTS-93 scale. Following preoperative consultations between patients and medical teams, the patients were divided into two groups: the 3D-printed group (n = 19), and the modular group (n = 13). No statistically significant differences were observed between the two groups with respect to age, gender, BMI values, tumor volume, tumor location, preoperative MSTS and VAS scores, or the duration of follow-up. The baseline characteristics of the patients are summarized in Table 1.

**Table 1:** Characteristics of patients undergoing hemipelvic replacement surgery

Characteristics	All patients	3D-printed group	Modular group	*P* value
Number	32	19	13	
Demographic				
Sex*				0.62
Male	18 (56.2)	10 (52.6)	8 (61.5)	
Female	14 (43.8)	7 (36.8)	7 (53.8)	
Age†(yr)	45.0 ± 13.4	46.3 ± 11.5	43.0 ± 16.0	0.50
BMI† (kg/m^2^)	24.4 ± 3.1	24.9 ± 2.9	23.8 ± 3.3	0.34
Follow-up time† (mo)	41.6 ± 10.5	42.3 ± 12.0	40.5 ± 8.0	0.64
Tumor histology*				0.47
Chondrosarcoma	12 (37.5)	7 (36.8)	5 (38.5)	
Osteosarcoma	7 (21.9)	4 (21.1)	3 (23.1)	
Ewing sarcoma	6 (18.8)	3 (15.8)	3 (23.1)	
Solitary plasmacytoma	2 (6.3)	2 (10.5)		
Synovial sarcoma	2 (6.3)	2 (10.5)		
Spindle cell carcinomas	1 (3.1)	1 (5.3)		
Myofibroblastic sarcoma	1 (3.1)		1 (7.7)	
Solitary fibrous tumor	1 (3.1)		1 (7.7)	
Tumor volume				
(Length × Width × Height, cm)				
Tumor length†(cm)	9.7 ± 1.6	10.1 ± 2.6	9.1 ± 1.6	0.22
Tumor width†(cm)	6.9 ± 1.2	6.9 ± 1.3	6.7 ± 1.2	0.80
Tumor height†(cm)	5.2 ± 1.2	5.6 ± 1.3	4.8 ± 1.1	0.06
Preoperative staging*				0.13
IIB	27 (90.6)	16 (84.2)	11 (84.6)	
III	5 (9.4)	3 (15.9)	2 (15.4)	
Neoadjuvant chemotherapy				
No. of patients*	14 (4.8)	8 (42.1)	6 (46.2)	
Enneking Reconstruction Classification*				0.87
Type I + II	8 (25.0)	5 (26.3)	3 (23.1)	
Type II + III	11 (34.4)	7 (36.8)	4 (30.8)	
Type I + II + III	13 (40.6)	7 (36.8)	6 (46.2)	
Preoperative MSTS Score	15.0 ± 2.4	15.5 ± 2.5	14.3 ± 2.0	0.18
Preoperative HHS Score	63.5 ± 6.6	64.1 ± 7.5	62.7 ± 5.3	0.58

*The values are given as the number of patients, with the percentage in parentheses

†The values are given as the mean and the standard deviation

### Custom-made and modular hemipelvic prostheses

- *Custom-made hemipelvic endoprostheses*: Designed by our clinical team and manufactured by Chunli Co., Ltd. (Tongzhou, Beijing, China), these prostheses are individually tailored to fit each patient’s pelvic defect shape with a bone-mimicking porous structure to facilitate bone ingrowth. Detailed information about the prostheses and specific design procedures can be found in previous report [38].
- *Modular hemipelvic prostheses*: Provided by Chunli Co., Ltd. (Tongzhou, Beijing, China). These prostheses comprise four components: Chunli System fixation device (CS fixator), pubic plate, acetabular cup, and polyethylene acetabular liner [43].

### Surgical techniques

All surgeries were performed by the same senior orthopedic surgeon. Patients in lateral decubitus position. Kocher-Langenbeck and Smith-Petersen approaches were combined, with optional inguinal extension [14]. Preserving hip joint muscles and their attachment points requires emphasis. For the 3D-printed group, precise osteotomy was assisted by cutting guides and validated with plastic prostheses. The prosthesis was implanted and fixed with multiple screws based on the preoperative plan. After pelvic reconstruction, the polyethylene liner angle within the acetabulum was appropriately adjusted (5°–10°) and fixed with bone cement. For the modular group, osteotomy was performed based on ensuring sufficient tumor margins while considering the actual situation. A suitable CS fixator was selected, and screws were fixed to the remaining sacroiliac bones. The acetabulum position was located using C-arm fluoroscopy, with an abduction angle of 45° and an anteversion angle of 15°–25°. If needed, the polyethylene cup angle was adjusted. In cases of tumor involvement in Zone III of the pelvis, an appropriate length of pubic plate was used for reconstruction. Finally, the installation of femoral head and neck prostheses and the reduction of the hip joint were carried out. The remaining muscles around the hip joint and their attachment points were subsequently sutured onto the prosthesis.

### Postoperative management

Within the first week after surgery, patients performed non-weight-bearing early rehabilitation exercises to enhance hip muscle strength and balance. Two rehabilitation exercise methods were used: (1) Active maintenance of the affected limb with 15°–25° hip abduction, 60°–80° hip flexion, and 90° knee flexion. (2) Active knee extension and maintenance with 15°–25° hip abduction, 20°–30° hip flexion, and 30°–45° knee flexion. The postoperative rehabilitation plan is as follows:

- *1–2 weeks post-surgery*: Transitioned to non-weight-bearing standing and hip flexion training.
- *2–4 weeks post-surgery*: Gradually increased weight-bearing training (starting from 10 kg) to match the healthy limb’s force.
- *After 4 weeks*: Encouraged hip abduction and extension exercises, assisted walking with aids.
- *First 3 months post-surgery*: Used T-shaped pillows and anti-rotational shoes for sleeping.
- *After 3 months*: Attempted walking without crutches, followed a one-week leg-crossing and squatting training. Achieved walking without crutches and hip flexion beyond 90°.

### Follow-up routine

The follow-up routine includes systematic clinical and radiological evaluations at 1, 2, and 3 months, every 3 months for the first 2 years, and then every 6 months. These evaluations are independently assessed by an unbiased surgeon. Specific indicators assessed include:

- *Surgical indicators*: Operation duration and blood loss. The bleeding is calculated by the anesthesiologist and surgical nurse.
- *Function*: Lower-limb function is assessed using the Musculoskeletal Tumor Society 93 (MSTS-93) and Harris hip score (HHS) at each follow-up.
- *Complications*: This includes monitoring for infection, local recurrence, dislocation, aseptic loosening, endoprosthetic breakage, and delayed wound healing.
- *Radiological outcome*: Osteointegration is assessed using Tomosynthesis Shimadzu Metal Artefact Reduction Technology (T-SMART). The implant-host bone interfaces are analyzed to quantify bone integration efficacy in all patients. Furthermore, the accuracy of acetabular component reconstruction in terms of the hip center of rotation within the hemipelvic prosthesis was assessed postoperatively using pelvic X-rays (Anteroposterior view). The acetabular eccentricity (medial–lateral) was defined as the distance between two lines: one drawn vertically from the center of the femoral head to the horizontal line and the other drawn vertically from the pubic symphysis to the same horizontal line. The acetabular eccentricity (superior-inferior) was defined as the vertical distance between two lines drawn perpendicular to the central axis of the hemipelvic prosthesis/pelvis within the acetabulum.

### Statistical analysis

Independent-samples Student’s t-test was used for normally distributed data, including operating time, intraoperative blood loss, HHS score, MSTS93 functional score, acetabular lever arm, and acetabular height. For non-normally distributed data, the Mann–Whitney U test was applied. Analysis was performed using SPSS 21.0 (IBM Corp., Armonk, NY). A p-value of less than 0.05 was considered statistically significant.

### Biomechanical study

#### Creation of 3D finite element models

**Fig. 1:**
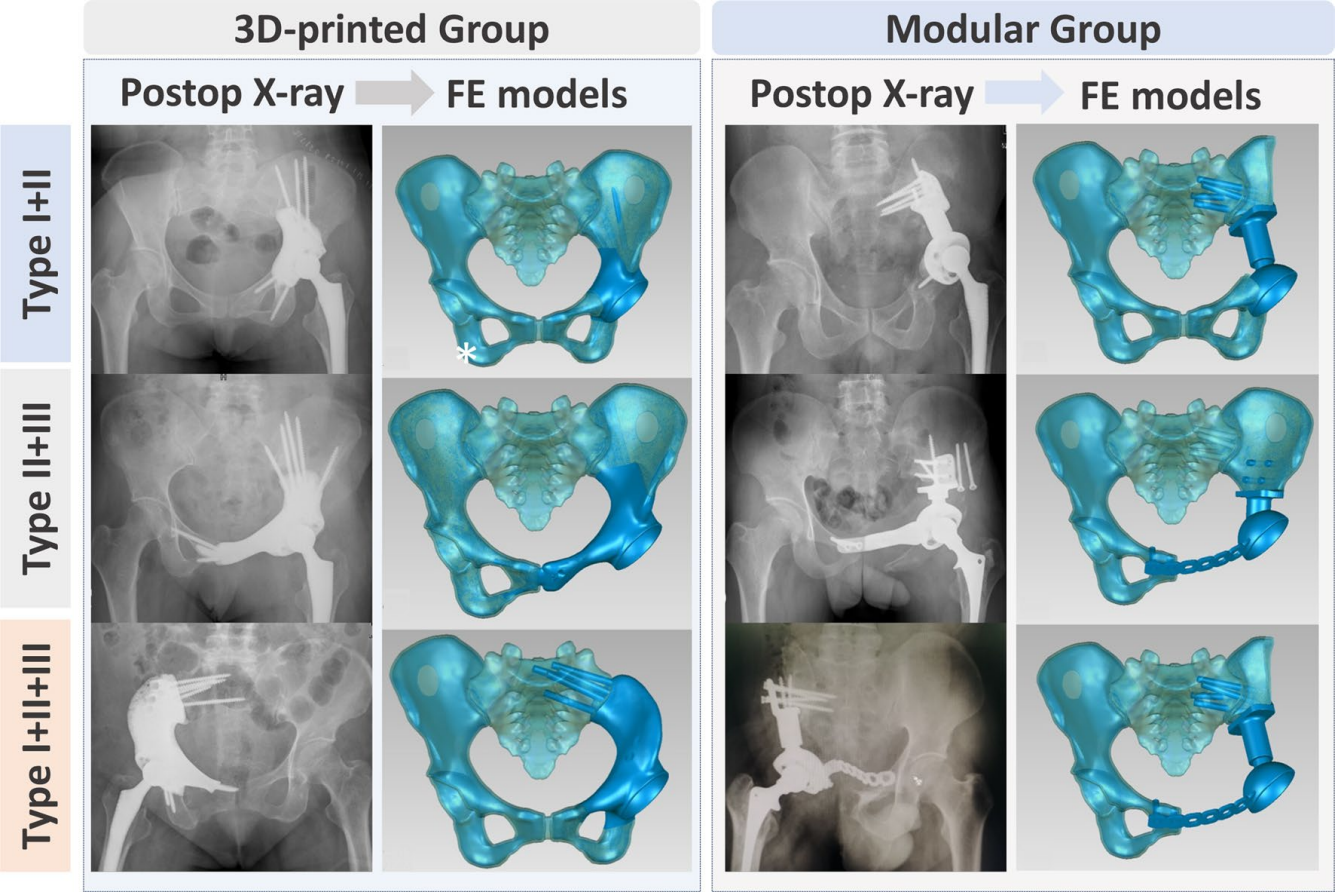
Three-Dimensional finite element models of pelvic defects post tumor resection and reconstruction: 3D FE models illustrating typical pelvic defects after acetabular tumor resection and reconstruction using 3D printing or modular hemipelvic prostheses. Key components (ilium, sacrum, prosthesis, and screws) are assembled in the models. The transformation from cortical bone to cancellous bone models is demonstrated through a global offset of "-2.0 mm." Internal cancellous bone regions are marked with asterisks (*)

We established 3D finite element models for normal pelvis and prosthetic reconstruction by first selecting CT data from a healthy adult volunteer (height = 170 cm, weight = 71 kg, The pelvic anatomical parameters closely approximate the median values of the corresponding parameters in the clinical study patients). After importing the data into Mimics V20.0 (Materialise Corp., Leuven, Belgium), we constructed a normal pelvic model, distinguishing between cortical and trabecular bone. Next, under the guidance of experienced surgeons, preoperative simulations (bone resection, prosthetic implantation, and screw fixation) were performed using 3D CT/MRI data from actual pelvic tumor patients, resulting in finite element models for prosthetic reconstruction. The 3D-printed prosthetic models followed previously established methods [38], while the 3D finite element models for modular prosthetic reconstruction were based on manufacturers’ specifications (Fig. 1).

#### Material assignment and mesh

In Abaqus 6.17 (Dassault Systèmes, Paris, France), all materials were assumed to be homogeneous, isotropic, and linearly elastic. Material properties, including elastic modulus and Poisson’s ratio, were assigned based on values from previous studies [17, 21, 44] (Table 2). For accurate geometry representation, tetrahedral elements with quadratic shape functions (C3D10) and displacement degrees of freedom were used to discretize the pelvis and implant. After meshing and optimization, the number of nodes and elements for each model is shown in Table 3.

**Table 2:** Material properties of the bone and implants

Materials	Modulus of elasticity (MPa)	Poisson’s ratio
Cortical bone	17,000	0.3
Cancellous bone	150	0.2
sacroiliac joint cartilage	54	0.4
pubic symphysis cartilage	5	0.45
Ti6Al4V	110,000	0.3

**Table 3:** Mesh division of each three-dimensional finite element model

3D Finite Element Model	The number of elements	the number of nodes
Normal pelvis	1,364,036	2,132,141
3D-printed endoprosthesis + type I + II pelvic defects	1,517,556	2,352,529
3D-printed endoprosthesis + type II + III pelvic defects	1,322,289	2,059,568
3D-printed endoprosthesis + type I + II + III pelvic defects	1,203,196	1,851,679
Modular endoprosthesis + type I + II pelvic defects	1,228,436	1,914,892
Modular endoprosthesis + type II + III pelvic defects	1,208,148	1,896,667
Modular endoprosthesis + type I + II + III pelvic defects	1,087,526	1,702,220

#### Loads and constraints

This study simulated physiological pelvic loading by applying 2/3 of the volunteer’s body weight vertically above the sacrum, specifically in alignment with the fifth lumbar vertebra [3]. By applying constraints to various anatomical regions of the pelvis, this study simulated four physiological postures, and constraints were set as follows:

Normal pelvic model:

- *Bipedal stance*: Both acetabula were constrained.
- *Unipedal stance*: One acetabulum was constrained.
- *Sitting*: Both ischial tuberosities were constrained.

**Fig. 2:**
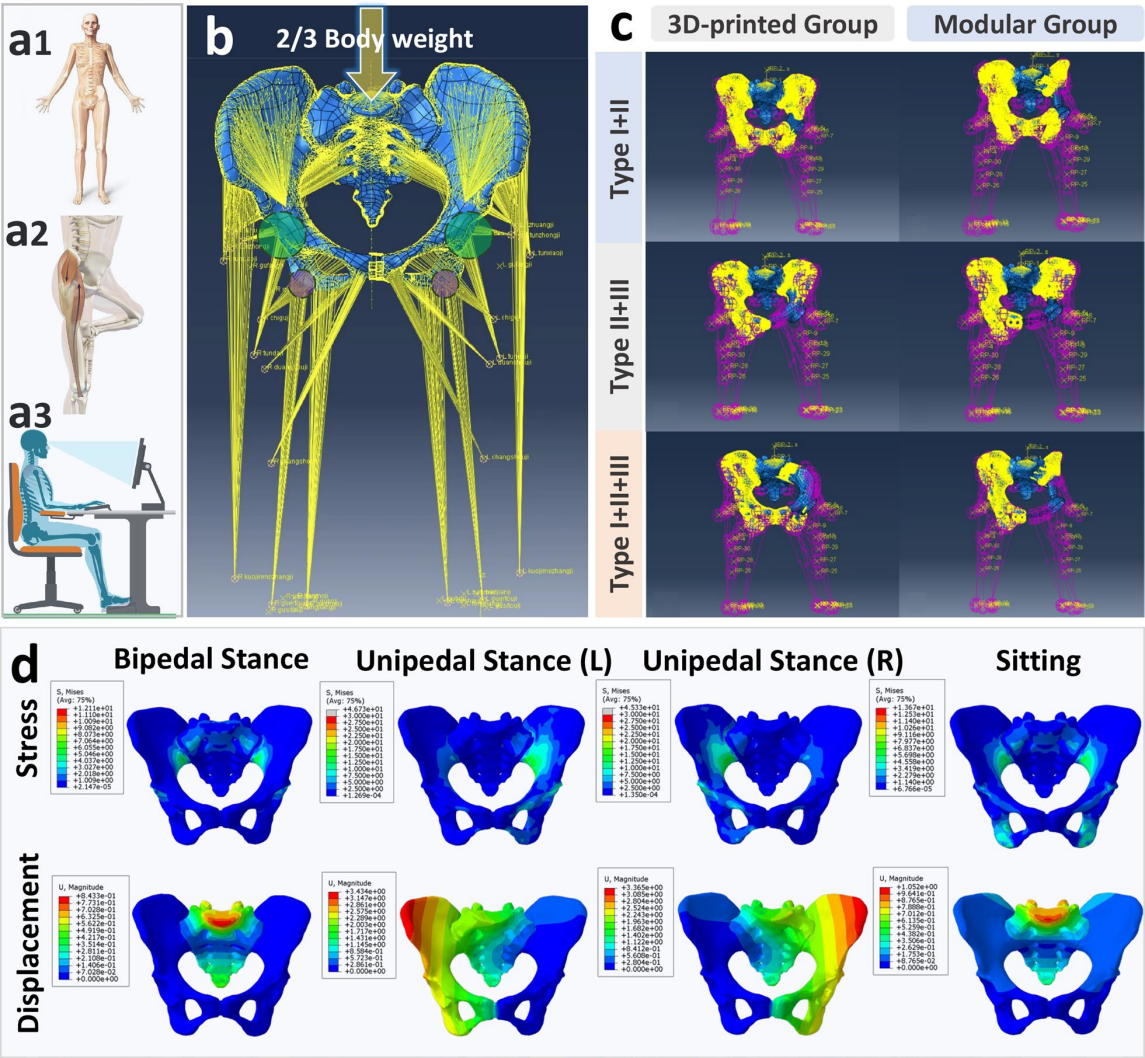
Loads and Constraints: **a** Simulated pelvic ring stress in three positions: **a1** bilateral standing, **a2** single-leg standing, and **a3** sitting after acetabular tumor resection and reconstruction. **b** Arrows represent the vertical stress applied to the sacrum, simulating 2/3 of the body weight, depicting different mechanical characteristics of the pelvic ring under different conditions. Green circles (purple circles) indicate the constrained regions at the acetabulum (ischial tuberosity). Yellow lines represent spring elements simulating the interaction between the pelvic region and lower limb muscles (accounting for muscle attachment points). **c** Muscle load illustration and mesh display in the 3D printing and modular groups: Demonstrating muscle load distribution in three types of pelvic defect reconstructions. Mesh representation shows the 3D printing and modular prosthetic components in place. **d** FEA results of stress and displacement distribution in a normal pelvic ring

Prosthetic-reconstructed pelvic model:

- *Bipedal stance*: Both acetabula were constrained.
- *Standing on the affected leg*: The acetabulum on the affected side was constrained.
- *Standing on the healthy leg*: The acetabulum on the healthy side was constrained.
- *Sitting*: Both ischial tuberosities were constrained, or in cases where the ischium was not reconstructed, the lower edge of the acetabulum was constrained. (Fig. 2a, b).

Furthermore, linear spring elements were used to simulate muscle forces with tension-only loading [28]. To create more realistic postoperative 3D finite element models with varying muscle strengths, muscle origin and insertion points were identified based on each patient’s postoperative 3D CT results (Soft tissue window setting) [17]. Muscle reconstruction ratios were calculated as the affected side’s muscle volume percentage compared to the healthy side (Additional file 1: Fig. S1). The 3D finite element model of the pelvis with prosthesis reconstruction was built using normal pelvic muscle loads (Table 4), and the stiffness of linear springs was adjusted to simulate muscle forces (Fig. 2c).

**Table 4:** Relevant muscles and stiffness values in normal pelvic model

Muscles	Stiffness (N/mm)	Number (N)
Gluteus maximus	344	4
Gluteus medius	779	8
Gluteus minimus	660	8
Iliacus	167	2
Rectus femoris	39	2
Tensor fasciae latae	13	4
Sartorius	92	2
Pectineus	306	2
Semitendinosus	44	2
Semimembranosus	100	2
Biceps femoris	74	2
Adductor magnus	257	4
Adductor longus	134	2
Adductor brevis	499	2
Gracilis	28	2
Piriformis	90	2
Quadratus Femoris	372	2

#### Finite element analysis

The study used finite element analysis to assess stress distribution and displacement in the pelvis under different positions and muscle attachment conditions for (1) The normal pelvic model; (2) The prosthetic-reconstructed pelvic model as a whole; (3) The prosthetic components and screws.

## Results

### FEM results

#### Stress and displacement distribution in normal pelvis

The normal pelvic model exhibits a stable, continuous, and evenly distributed stress pattern in different positions, with no significant stress concentration areas observed (Fig. 2d). Specifically, during bipedal stance, stress was symmetrically transmitted through the sacroiliac joints and then transitions along the arcuate lines and greater sciatic notches to the acetabula, peaking at 12.11 MPa. The left unipedal stance showed stress transmission to the left acetabulum, peaking at approximately 20 MPa, while the right unipedal stance exhibited a symmetrically opposite stress pattern. In sitting, stress was symmetrically transmitted through the sacroiliac joints to the ischial tuberosities, with a peak of 13.67 MPa. The overall displacement is symmetric, with the maximum displacement being 0.8433 mm during bipedal stance, 3.434 mm during the left unipedal stance, and 1.052 mm during sitting.

#### Stress and displacement distribution in pelvic reconstruction with corresponding implants

**Fig. 3:**
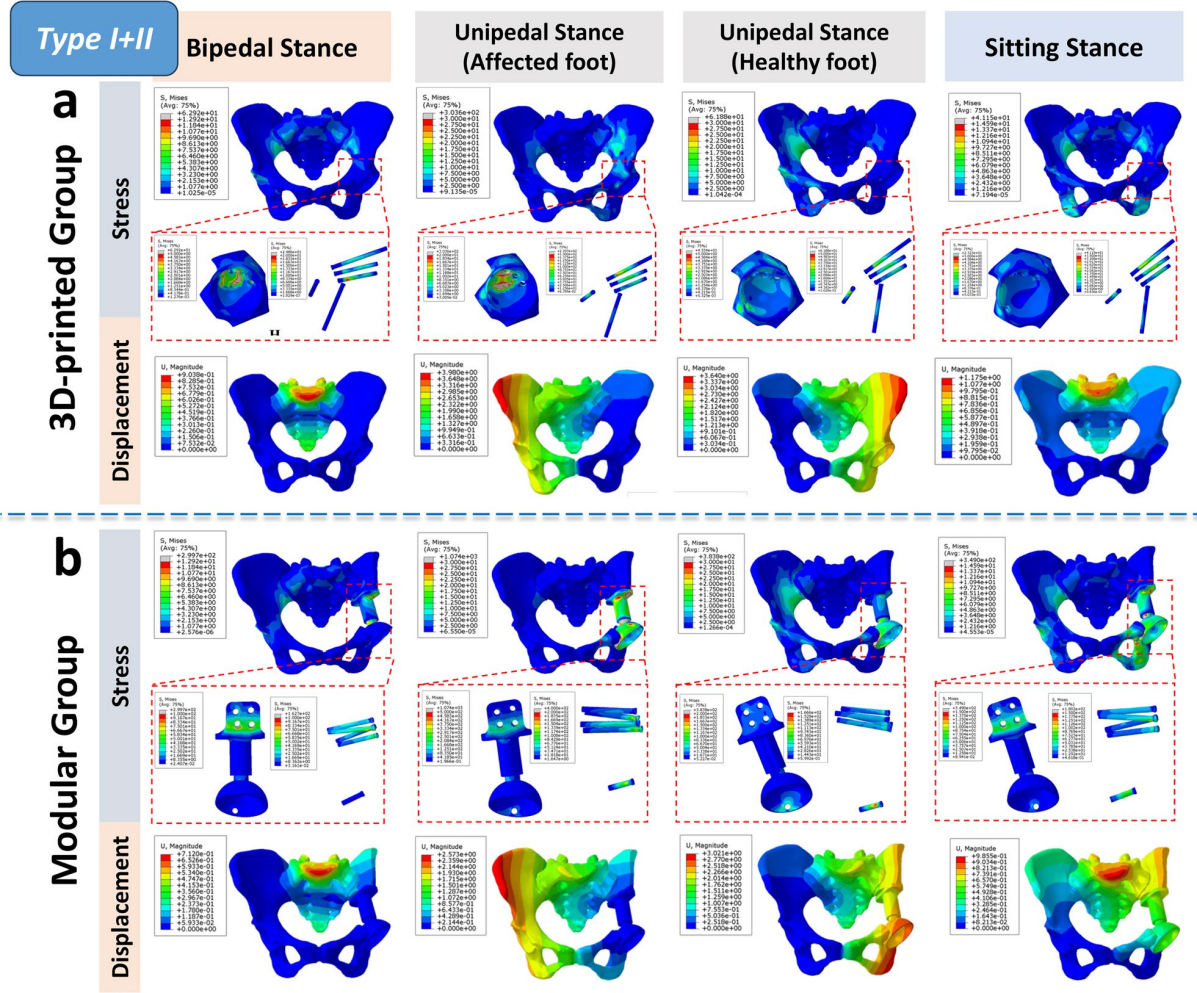
Biomechanical differences in pelvic ring reconstruction after Type I + II resection: Comparing different reconstruction methods using 3D-printed hemipelvic endoprostheses (**a**) and modular hemipelvic endoprostheses (**b**) in four common physiological postures, the stress distribution is more uniform and gradual in the 3D-printed prosthetic reconstruction. However, in the modular prosthetic reconstruction, significant stress concentration is evident

**Fig. 4:**
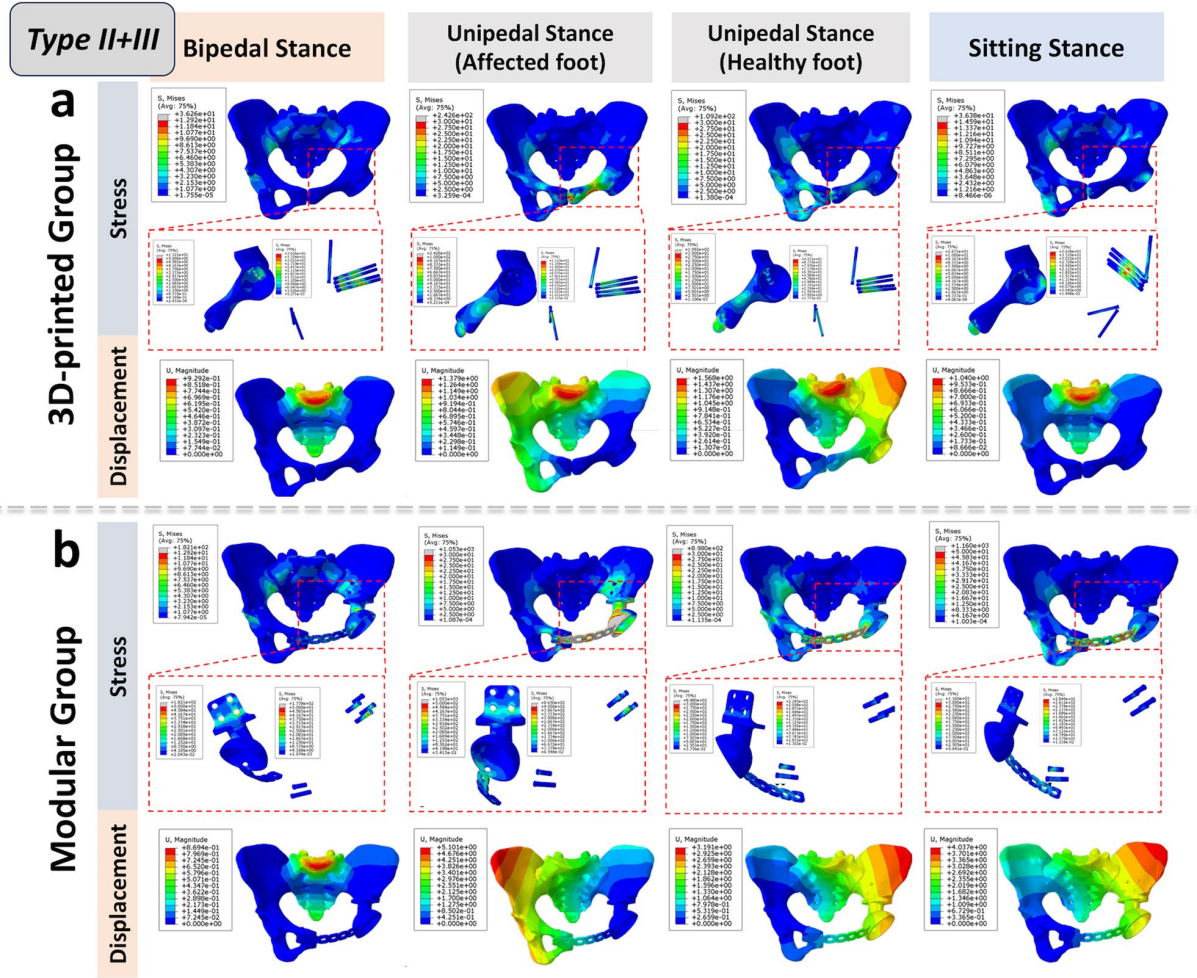
Biomechanical differences in pelvic ring reconstruction after Type II + III resection: Comparing different reconstruction methods using 3D-printed hemipelvic prostheses (**a**) and modular hemipelvic prostheses (**b**) in four common physiological postures, the 3D-printed prosthetic reconstruction exhibits a more uniform and gradual stress and displacement distribution. Conversely, the modular prosthetic reconstruction shows significant stress concentration at the pubic symphysis region

**Fig. 5:**
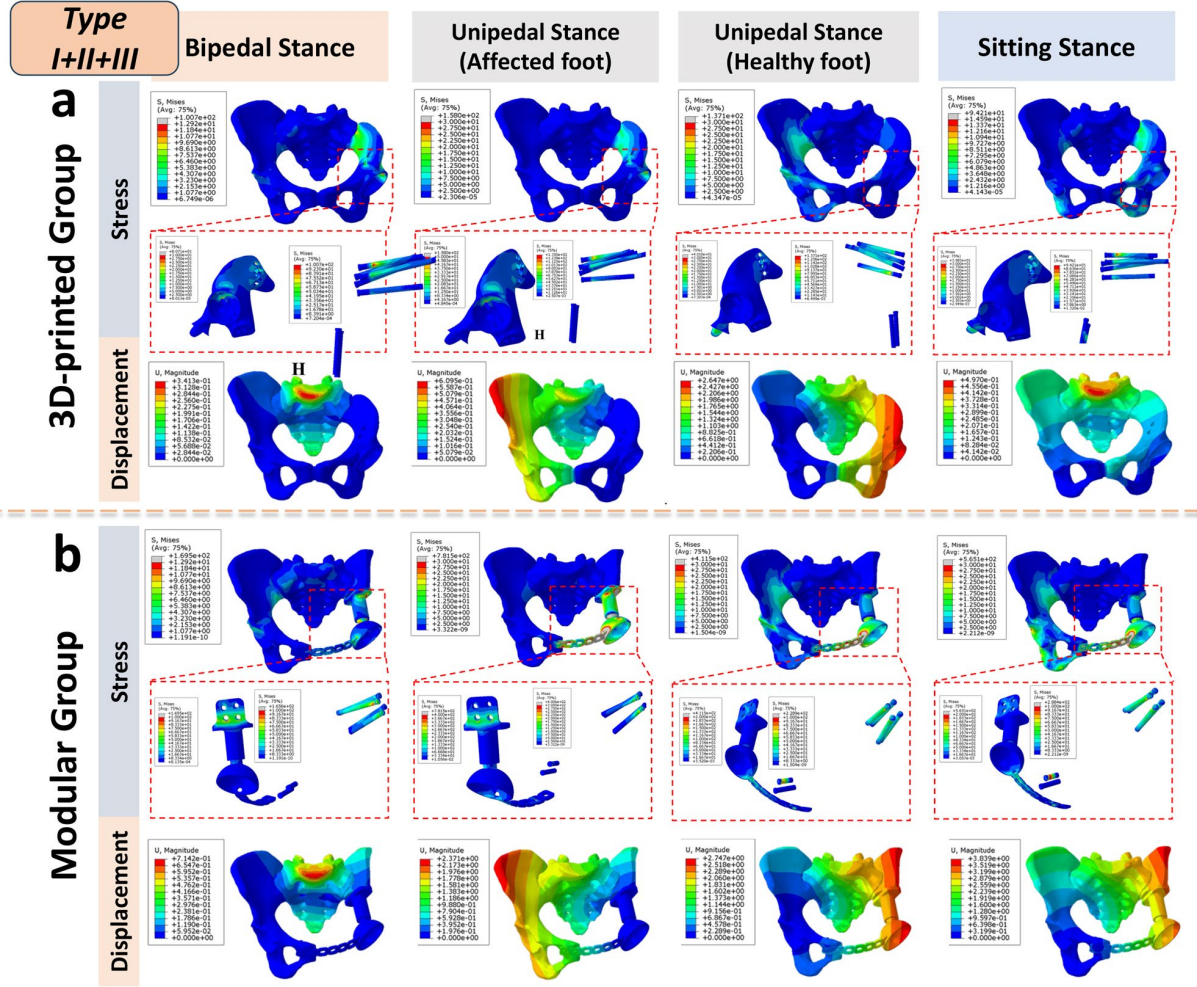
Biomechanical differences in pelvic ring reconstruction after Type I + II + III resection: Comparing different reconstruction methods using 3D-printed hemipelvic prostheses (**a**) and modular hemipelvic prostheses (**b**) in four common physiological postures, the 3D-printed prosthetic reconstruction exhibits a more uniform and natural stress and displacement distribution, closely resembling a normal pelvis. However, the modular hemipelvic prosthesis shows evident stress concentration at the pubic symphysis and CS fixator regions

Overall, the 3D-printed group exhibited a more uniform, continuous, and gentle stress distribution pattern in various bone defect reconstructions, resembling the physiological stress distribution observed in the normal pelvic model under muscle loading in the finite element analysis (FEA) results. Conversely, the modular group displayed a stiffer and discontinuous stress distribution, with noticeable stress concentrations, particularly in the CS fixator and certain screws, at times surpassing the yield stress of titanium alloy, markedly deviating from the FEA results of the normal pelvic model (Figs. 3, 4, 5). Detailed information on the highest stress, displacement, and their respective locations in different regions of the normal pelvis, 3D-printed monolithic, and group-assembly prosthetic-reconstructed pelvic models for tumor-related bone defects can be found in Table 5.

**Table 5:** Peak stress and displacement distribution in 3D finite element models of reconstructed pelvises

Types of prostheses	Enneking resection type	Bilateral standing position		Affected foot (left) single-leg standing position		Healthy foot (right) single-leg standing position		Seated position	
		Location of highest stress /(Mpa)	Location of maximum displacement /(mm)	Location of highest stress /(Mpa)	Location of maximum displacement /(mm)	Location of highest stress /(Mpa)	Location of maximum displacement /(mm)	Location of highest stress /(Mpa)	Location of maximum displacement /(mm)
–	Normal pelvis	Bilateral Arcuate line/12.11	Upper part of the sacrum /0.84	Upper part of the left acetabulum /46.73	Right Anterior Superior Iliac Spine /3.43	Upper part of the right acetabulum /45.33	Left Anterior superior iliac spine/3.37	Ischial tuberosity/13.67	Upper part of the sacrum /1.05
3D-printed endoprosthesis	Type I + II	Bottom of the acetabular cup /62.92	Upper part of the sacrum /0.85	Bottom of the acetabular cup /303.6	Right Anterior Superior Iliac Spine /3.75	Iliac screw pathway /45.34	Left Anterior superior iliac spine/3.43	Iliac screw pathway /23.23	Upper part of the sacrum /1.10
	Type II + III	Sacroiliac screw pathway /13.21	Upper part of the sacrum /0.87	Pubic symphysis plate /242.6	Upper part of the sacrum /1.30	Prosthesis-pubic bone contact area /109.2	Upper part of the sacrum /1.47	Bottom of the acetabular cup /26.71	Upper part of the sacrum /0.98
	Type I + II + III	Sacroiliac screw pathway /80.71	Upper part of the sacrum /0.33	Bottom of the acetabular cup /158.0	Right Anterior Superior Iliac Spine /0.58	Prosthesis-pubic bone contact area /40.10	Outer part of the acetabular cup /2.53	Sacroiliac screw pathway /39.83	Upper part of the sacrum /0.47
Modular endoprosthesis	Type I + II	CS internal fixator /299.7	Upper part of the sacrum /0.71	CS internal fixator/1074	Right Anterior Superior Iliac Spine /2.57	Pubic screw/383.8	Left ischial tuberosity/3.02	CS internal fixator/349.0	Upper part of the sacrum /0.99
	Type II + III	CS internal fixator /182.1	Upper part of the sacrum /0.87	Pubic symphysis plate /1053	Right Anterior Superior Iliac Spine /5.10	Pubic symphysis plate /898.0	Left Anterior superior iliac spine/3.19	Pubic symphysis plate /1160.0	Anterior superior iliac spine/4.03
	Type I + II + III	CS internal fixator /169.5	Upper part of the sacrum /0.71	CS internal fixator /781.5	Right Anterior Superior Iliac Spine /2.37	Pubic symphysis plate /411.5	Outer part of the acetabular cup /2.74	Pubic symphysis plate /565.1	Anterior part of the ilium/3.84

### Clinical outcomes

#### Demographics

This study involved 32 patients with primary malignant acetabulum tumors (18 males, 14 females). The average follow-up time for all patients was 41.6 ± 10.5 months. 19 patients received 3D-printed custom hemipelvic prostheses, with an average follow-up time of 42.3 ± 12.0 months, while 13 patients received modular prostheses, with an average follow-up time of 40.5 ± 8.0 months. Osteosarcoma and Ewing sarcoma patients had neoadjuvant chemotherapy, followed by post-surgery chemotherapy after incision healing. Radiotherapy was avoided to support wound healing.

#### Surgical outcomes

The 3D group exhibited significantly higher blood loss [2121.1 ± 686.8 ml (range, 1000.0–3500.0 ml) vs 1600.0 ± 505.0 ml (range, 1000.0–2500.0 ml), *p* < 0.05] and longer surgical time [426.2 ± 67.0 min (range, 300.0–592.0 min) vs 301.7 ± 48.6 min (range, 243.0–410.0 min), *p* < 0.05] compared to the modular group.

#### Functional assessment

The 3D group exhibited significantly higher mean Musculoskeletal Tumor Society (MSTS) scores compared to the modular group [24.3 ± 1.8 (range, 21.0–27.0) vs 21.8 ± 2.0 (range, 15.0–26.0), *p* < 0.05] and mean Harris Hip Score (HHS) [79.8 ± 5.2 (range, 72.0–85.0) vs 75.3 ± 3.5 (range, 70.0–82.0), *p* < 0.05] during the last follow-up, indicating improved hip joint function in the 3D group (Additional file 2: Fig. S2).

#### Complications

Intraoperative complications were absent. Postoperative complications occurred in 11 patients: 3 cases (26.3%) in the 3D group and 6 cases (46.2%) in the modular group. Poor wound healing was the most common postoperative complication among all patients.

In the 3D group:

- One had an upper sacroiliac joint screw fracture (5.3%) one year postoperatively but remained asymptomatic without affecting function. (Fig. 3Sa).
- One exhibited distal-bone interface loosening (5.3%) and screw fractures 3 years postoperatively, which improved with revision surgery (Fig. 3Sb).
- Two cases of poor wound healing (10.5%) resolved with intensive wound dressing.
- One patient (5.3%) experienced postoperative hip dislocation two days after surgery, which was promptly addressed through closed reduction. Stability was maintained using a T-shaped pillow and anti-rotation shoes (Additional file 3: Fig. S3c).

In the modular group:

- Two cases had poor wound healing (15.4%). One case healed with enhanced wound dressing, while another required debridements and negative pressure Vacuum Assisted Closure (VAC) system therapy to achieve healing.
- A deep prosthesis infection impacted one case (7.7%) and required extensive treatment one month after the surgery.
- Three cases (23.1%) encountered screw loosening, with one being managed conservatively two years after surgery, and the other two undergoing successful 3D-printed hemipelvic revision surgery, one at 1 year post-surgery and the other at 2.5 years post-surgery. (Additional file 3: Fig. S3d).

#### Radiological outcome

**Fig. 6:**
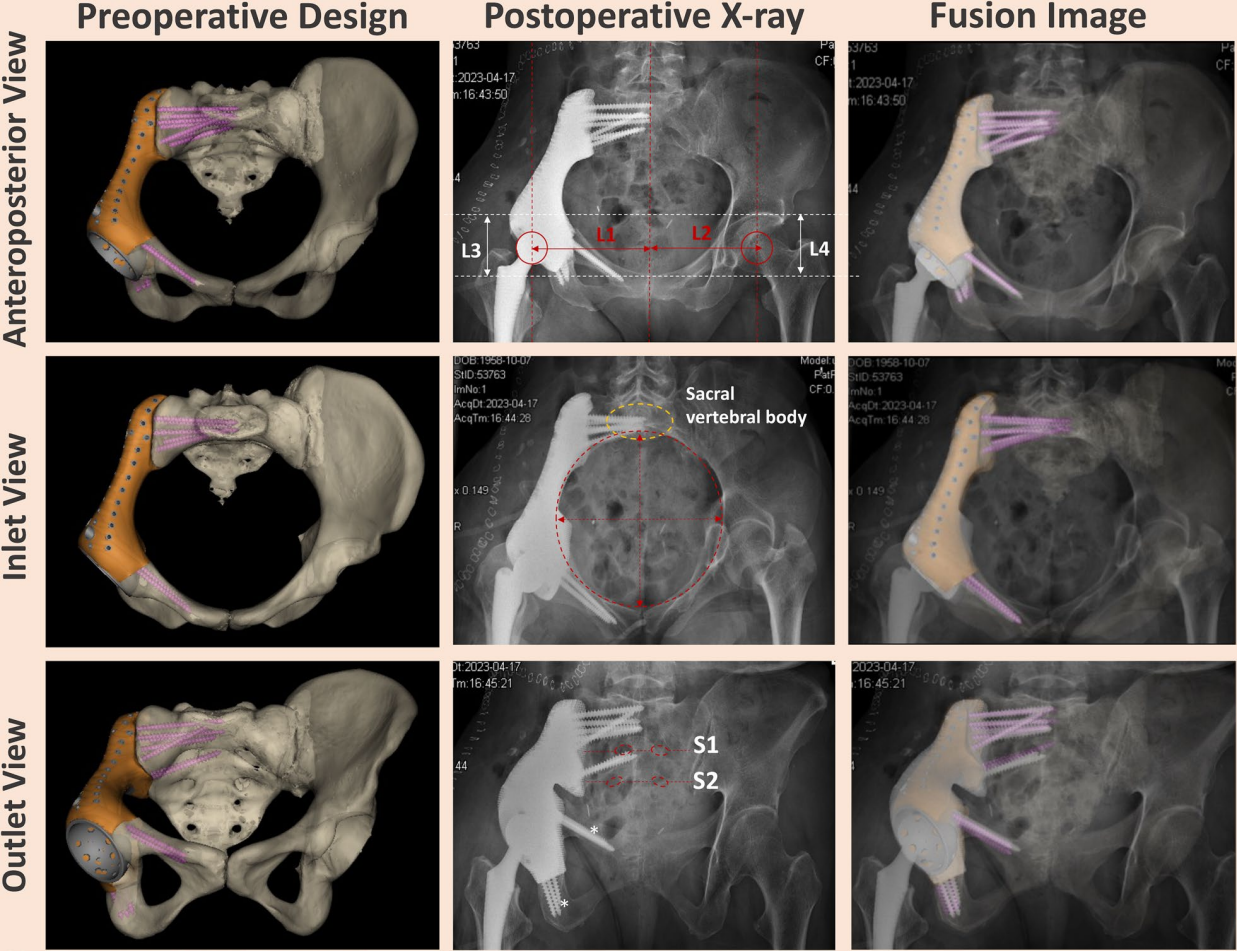
Illustrates postoperative multi-directional pelvic X-ray assessment, demonstrating precise execution of the following: Precise osteotomy, prosthesis placement, and multi-level screw fixation. Anteroposterior views of the pelvis reveal an accurate hip joint rotation center (indicated by red circle) and precise femoral head eccentricity in horizontal (L1 = L2) and vertical (L3 = L4) directions. The inlet view displays accurately positioned sacroiliac joint screws in the anterior–posterior direction, carefully avoiding the posterior vertebral canal (highlighted by the yellow circle, representing the safe vertebral region). Complete small pelvic ring reconstruction is indicated by the red dashed circle. The outlet view shows accurate sacroiliac joint screw placement, avoiding S1/S2 sacral foramina (indicated by the red dashed box). Ischium and pubic bone screws are precisely positioned for enhanced stability and support

**Fig. 7:**
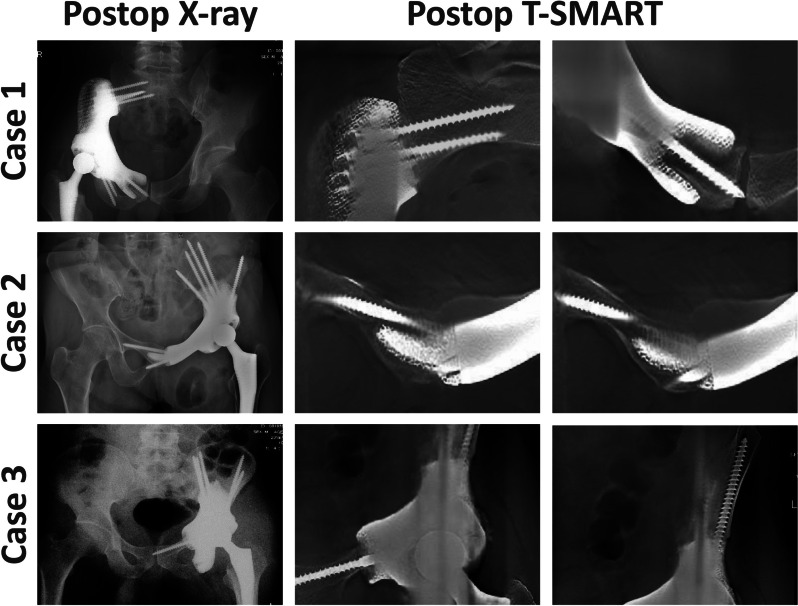
Successful prosthesis-bone integration using 3D-printed hemipelvic endoprosthetic reconstruction: In the last follow-up, T-SMART imaging confirmed effective osseointegration at the bone-implant interface

In the 3D printing group, all patients underwent precise osteotomy, accurate prosthesis implantation, and planned screw fixation based on preoperative simulated surgery. The acetabular eccentricity (medial–lateral) on the healthy side measured 94.7 ± 4.3 mm (range, 91.5.0–97.1 mm), and on the affected side, it was 93.7 ± 5.3 mm (range, 91.2.0–97.5 mm) (Fig. 6). Similarly, the acetabular eccentricity (superior-inferior) on the healthy side was 54.3 ± 4.7 mm (range, 50.8–57.2 mm), and on the affected side, it was 55.6 ± 4.8 mm (range, 52.2.0–58.3 mm). The comparison between healthy and affected sides showed no statistically significant differences (*p* > 0.05), indicating accurate hip joint reconstruction. During the final follow-up, 97.1% of patients (66/68) exhibited successful osseointegration of their implants (Fig. 7), with only one case experiencing distal prosthesis loosening (Additional file 3: Fig. S3b).

In the modular group, there was no effective bone integration at the prosthesis-bone contact. The healthy side acetabular eccentricity (medial–lateral) was 91.5 ± 5.8 (range, 89.0–94.0 mm), and on the affected side, it was 86.2 ± 9.0 mm (range, 64.0–99.0 mm). The healthy side acetabular eccentricity (superior-inferior) was 54.5 ± 5.8 mm (range, 50.2–57.2 mm), and on the affected side, it was 60.6 ± 6.0 mm (range, 55.3–65.0 mm). When comparing the healthy and affected sides, the differences in both acetabular eccentricity (medial–lateral) and acetabular eccentricity (superior-inferior) are statistically significant (*p* < 0.01). Moreover, postoperative X-rays revealed complications in some cases: 1 case showed inward bending of the pubic bone plate (Additional file 3: Fig. S3e), 1 case exhibited inward displacement of the acetabular cup (Additional file 3: Fig. S3f), and 2 cases had loose screws in the CS fixator area.

## Discussion

In recent years, pelvic prostheses have gradually emerged as the primary method for reconstructing the pelvic region following the removal of malignant or invasive tumors due to their initial stability, high acceptance of their appearance, and relatively swift functional recovery. Modular and 3D-printed integrated prostheses, in particular, have gained significant attention. One significant difference between these two reconstruction methods lies in the fact that modular hemipelvic prostheses often do not encompass a complete reconstruction of the pelvic ring, while 3D-printed integrated prostheses have the capability to fully restore the anatomical shape of the pelvic ring during the recovery period. This dissimilarity results in markedly different stress distribution patterns within the pelvic ring, which theoretically could have varying effects on the mid-term stability and lifespan of the prostheses. Understanding the distribution of stress within the pelvic ring and the principles of its transmission under normal physiological conditions is of paramount importance. It provides essential guidance for surgeons tasked with reconstructing the pelvic ring after the resection of periacetabular tumors.

Based on the finite element analysis (FEA) results of our study, the normal pelvis, under physiological stress, follows a stress transmission pathway from the spine to the sacroiliac joint, the pelvic ring, and ultimately to the hip joint. It’s worth noting that the posterior pelvic ring plays a crucial role in carrying the load. However, it’s important not to underestimate the contribution of the anterior pelvic ring in enhancing stability and preventing lateral spreading. These findings are consistent with previous biomechanical analyses of the pelvis, such as those by Tile and others [31, 32], who suggested that the anterior and posterior structures contribute 40% and 60%, respectively, to the overall stability of the pelvic girdle. According to their “suspension bridge concept”, the posterior pelvic ring primarily bears the main load transfer, while the anterior ring acts like a pull bar (strut), similar to a suspension bridge, enhancing stability and preventing lateral spreading [33]. Furthermore, regarding the stress distribution in pelvic ring prosthesis reconstruction, for all types of resections (type I + II/II + III/I + II + III resections), the 3D-printed group consistently exhibits stress distribution patterns closer to those of a normal pelvis. Stress peaks and displacement amplitudes are consistently lower in the 3D-printed group compared to the modular group. These findings suggest that this reconstruction method carries a lower risk of mechanical failure, such as screw fracture or loosening, and provides improved post-reconstruction stability [13, 16].

Modular prostheses, despite fully reconstructing the pelvic ring, have a major drawback of relatively high postoperative loosening rates [36]. This issue can, in significant part, be attributed to the unnatural stress transmission pattern that emerges after hemipelvic reconstruction using modular prostheses. The stress distribution, exerting both pushing and pulling forces on the implant from two sides, is likely one of the key factors contributing to the heightened risk of loosening. Moreover, during reconstruction, achieving a satisfactory acetabular location and orientation often requires sacrificing the fit at the anchor part [10]. Our FEA results showed that this poor prosthesis-pelvic match leads to multiple stress concentrations in modular hemipelvic prostheses. For example, the pubic plate part experiences significant stress concentration, coinciding with the observed bending deformity of the pubic plate in one patient during clinical follow-up. Another significant factor contributing to the loosening of modular endoprostheses may be incomplete osseointegration. In this study, the high-stress concentration in the CS fixation area of the modular prosthesis, combined with the lack of a porous surface structure, hindered osteointegration, making this area a common site for loosening. Even with the incorporation of porous structures onto the surface of modular implants to augment their integration, the fundamental design incongruity with the stress propagation dynamics of the pelvic ring persists [9, 23]. Consequently, the precise manipulation of the hip joint's rotational center during surgical intervention remains challenging, which in turn may engender subsequent displacement under the influence of external forces during the extended postoperative period.

On the contrary, the 3D printing group achieved accurate bone resection with custom cutting guides, ensuring precise matching between the custom prosthesis and bone defect. FEA results demonstrated that the stress transmission in the reconstructed pelvic ring of this group closely resembled that of a normal pelvis. The stable initial reconstruction, along with the porous surface structure facilitating bone integration, further enhanced its mid-term stability. Consequently, the 3D printing group achieved satisfactory functional outcomes (average MSTS score of 76.7%), surpassing other investigations [5, 11, 22, 23]. However, achieving better postoperative functionality with 3D-printed hemipelvic prostheses comes with some trade-offs, such as longer design and production time, and higher costs. These may gradually decrease with wider adoption and market availability of the technology. The main concern lies in the ability to achieve precise preoperative planning and implantation. This demands utmost accuracy throughout the entire process, from preoperative cutting guides and prosthesis design to intraoperative bone resection, prosthesis implantation, and screw fixation. When contrasted with the flexible, convenient, and rapid implantation process of modular prostheses, it becomes understandable why the follow-up results reveal a somewhat longer operation time and slightly increased blood loss during surgery in the 3D printing group. Nevertheless, it's reassuring that these differences remain within acceptable ranges [23, 35, 45].

## Conclusion

Modular and 3D-printed hemipelvic endoprostheses are commonly used for limb-salvage reconstruction after periacetabular tumor resection. Modular ones offer easy implantation and flexibility but have a higher loosening rate due to poor prosthesis-host bone matching. 3D-printed hemipelvic endoprostheses, customized for individuals, precisely restore pelvic ring anatomy, improving fit and alignment. Compared to modular prostheses, it offers a closer approximation of normal pelvic physiological stress transmission. Mid-term clinical follow-ups have shown improved functional outcomes and bone integration. However, this technique demands high expertise, time, and effort for prosthesis fabrication. Surgical implantation is more challenging and may lead to longer operating times and increased intraoperative blood loss as potential drawbacks. Surgeons must weigh postoperative functional outcomes, complexity, and patient condition to decide the best treatment.

### Supplementary Information

**Additional file 1**. **Fig. S1**. Muscle Reconstruction Proportion Assessment Method: During surgery procedures, the extent of muscle resections varies among different muscle groups due to tumor involvement in specific regions. Postoperatively, the 3D CT measurement of the pelvic region includes the complete origin and insertion points of major hip joint functional muscle groups (adductor, abductor, and flexor muscles), represented by the longest dimensions (length, width, height) of each muscle group in the pelvic 3D CT. The product of these dimensions reflects the muscle content, and the ratio of affected side muscle volume to the healthy side is calculated, defining the muscle reconstruction rate. Based on the muscle reconstruction parameters, the stiffness of different regions in the pelvis with bone defects and different prosthetic reconstructions is proportionally adjusted to approximate the model to the real physiological state after hemipelvic prosthesis reconstruction.

**Additional file 2**. **Fig. S2** Functional Follow-up Photographs: The supplement figure illustrates functional follow-up photographs of patients who underwent pelvic ring reconstruction using 3D-printed and modular hemipelvic prostheses following tumor resection. The photographs depict the hip flexion function at 42 months post-surgery for three representative patients. In each patient's image, the left photograph captures the affected side during hip flexion in a standing position, while the right photograph portrays the healthy side during hip flexion in a standing position. These functional follow-up images provide valuable insights into the postoperative outcomes and the effectiveness of the two different hemipelvic prosthesis reconstruction techniques in restoring hip joint functionality after tumor resection.

**Additional file 3**. **Fig. S3** Typical postoperative complications in 3D-printed prosthetic hip reconstruction surgery: **a** Screw Fracture: Day 2 Postoperative X-ray (**a1**). One Year Postoperative X-ray (a2) displays a screw fracture (a2) at the uppermost part of the sacroiliac joint, evident by the red mark. Notably, the patient remained asymptomatic, and conservative observation was chosen. **b** Aseptic loosening: Postoperative 1-year Pelvic X-ray (**b1**) reveals loosening and fracture of the ischial screw. Postoperative 2-year X-ray (**b2**) shows loosening at the prosthesis-bone interface and multiple screw failures. **c** Hip Dislocation: Three Days Postoperative X-ray (**c1**) reveals hip dislocation. Successful closed reduction under general anesthesia was performed (**c2**). **d** Design and Application of 3D-Printed Hemipelvic Endoprosthesis for Revision of Aseptic Loosening: Preoperative Simulation **d1**, **d2** Depicted endoprosthesis migration and fractured screws. Illustrations **d3**, **d4** Demonstrated design of 3D-printed custom hemipelvic endoprosthesis and screw fixation. Pre-revision Radiographs **d5**, **d6** Displayed aseptic loosening, screw fracture, and endoprosthesis migration. Post-implantation Radiographs **d7**, **d8** Revealed successful reconstruction with custom hemipelvic endoprosthesis. **e** Three Years Post-Surgery: Pelvic X-ray showed suboptimal integration at modular hemipelvic endoprosthesis interface and inward acetabular cup movement. **f** Five Years Post-Surgery: Pelvic X-ray indicated inadequate integration at modular hemipelvic endoprosthesis interface, with pubic plate deformation and bending. (Reprinted with permission from Ref [68] ©2021 BMC Surgery).

## Data Availability

The datasets used and/or analysed during the current study available from the corresponding author on reasonable request.
