# Supplementary figures and images for: Biomechanical and clinical outcomes of 3D-printed versus modular hemipelvic prostheses for limb-salvage reconstruction following periacetabular tumor resection: a mid-term retrospective cohort study
Source: J Orthop Surg Res. 2024 Apr 23;19:258. doi: 10.1186/s13018-024-04697-w (PMC11040966; doi:10.1186/s13018-024-04697-w)

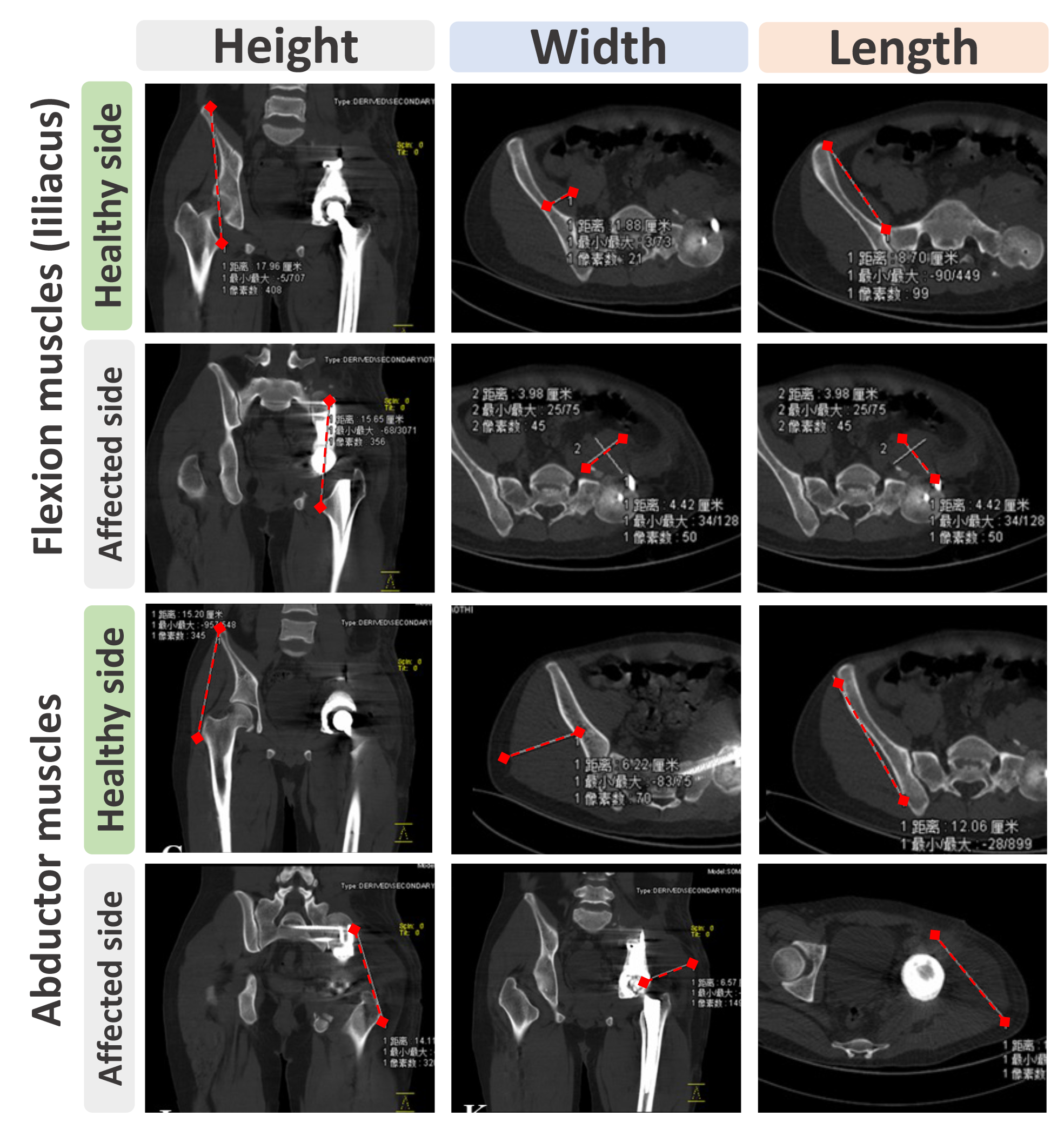

Supplement: Supplementary file 1 — Additional file 1. Fig. S1. Muscle Reconstruction Proportion Assessment Method: During surgery procedures, the extent of muscle resections varies among different muscle groups due to tumor involvement in specific regions. Postoperatively, the 3D CT measurement of the pelvic region includes the complete origin and insertion points of major hip joint functional muscle groups (adductor, abductor, and flexor muscles), represented by the longest dimensions (length, width, height) of each muscle group in the pelvic 3D CT. The product of these dimensions reflects the muscle content, and the ratio of affected side muscle volume to the healthy side is calculated, defining the muscle reconstruction rate. Based on the muscle reconstruction parameters, the stiffness of different regions in the pelvis with bone defects and different prosthetic reconstructions is proportionally adjusted to approximate the model to the real physiological state after hemipelvic prosthesis reconstruction. [file 13018_2024_4697_MOESM1_ESM.png]

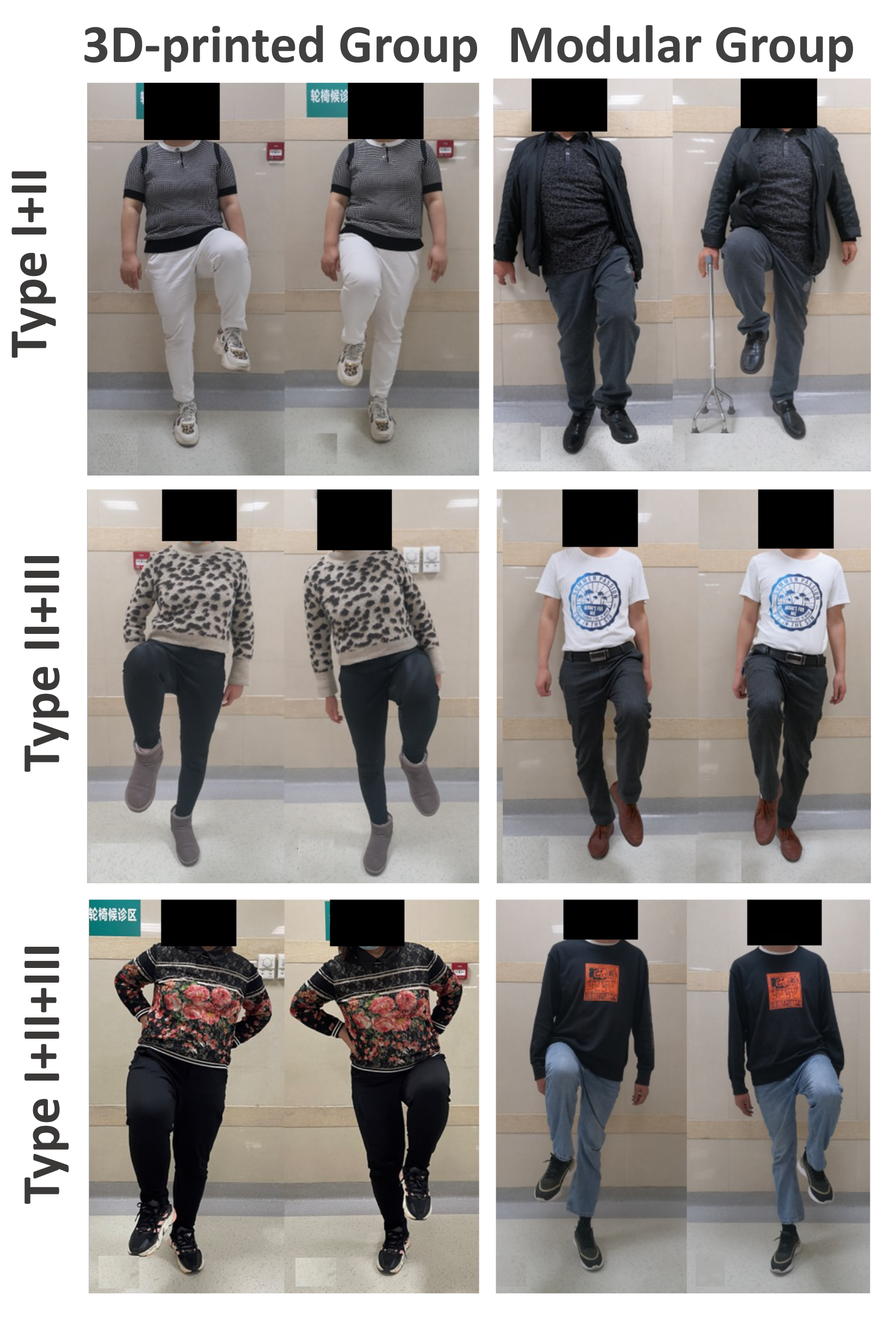

Supplement: Supplementary file 2 — Additional file 2. Fig. S2 Functional Follow-up Photographs: The supplement figure illustrates functional follow-up photographs of patients who underwent pelvic ring reconstruction using 3D-printed and modular hemipelvic prostheses following tumor resection. The photographs depict the hip flexion function at 42 months post-surgery for three representative patients. In each patient's image, the left photograph captures the affected side during hip flexion in a standing position, while the right photograph portrays the healthy side during hip flexion in a standing position. These functional follow-up images provide valuable insights into the postoperative outcomes and the effectiveness of the two different hemipelvic prosthesis reconstruction techniques in restoring hip joint functionality after tumor resection. [file 13018_2024_4697_MOESM2_ESM.png]

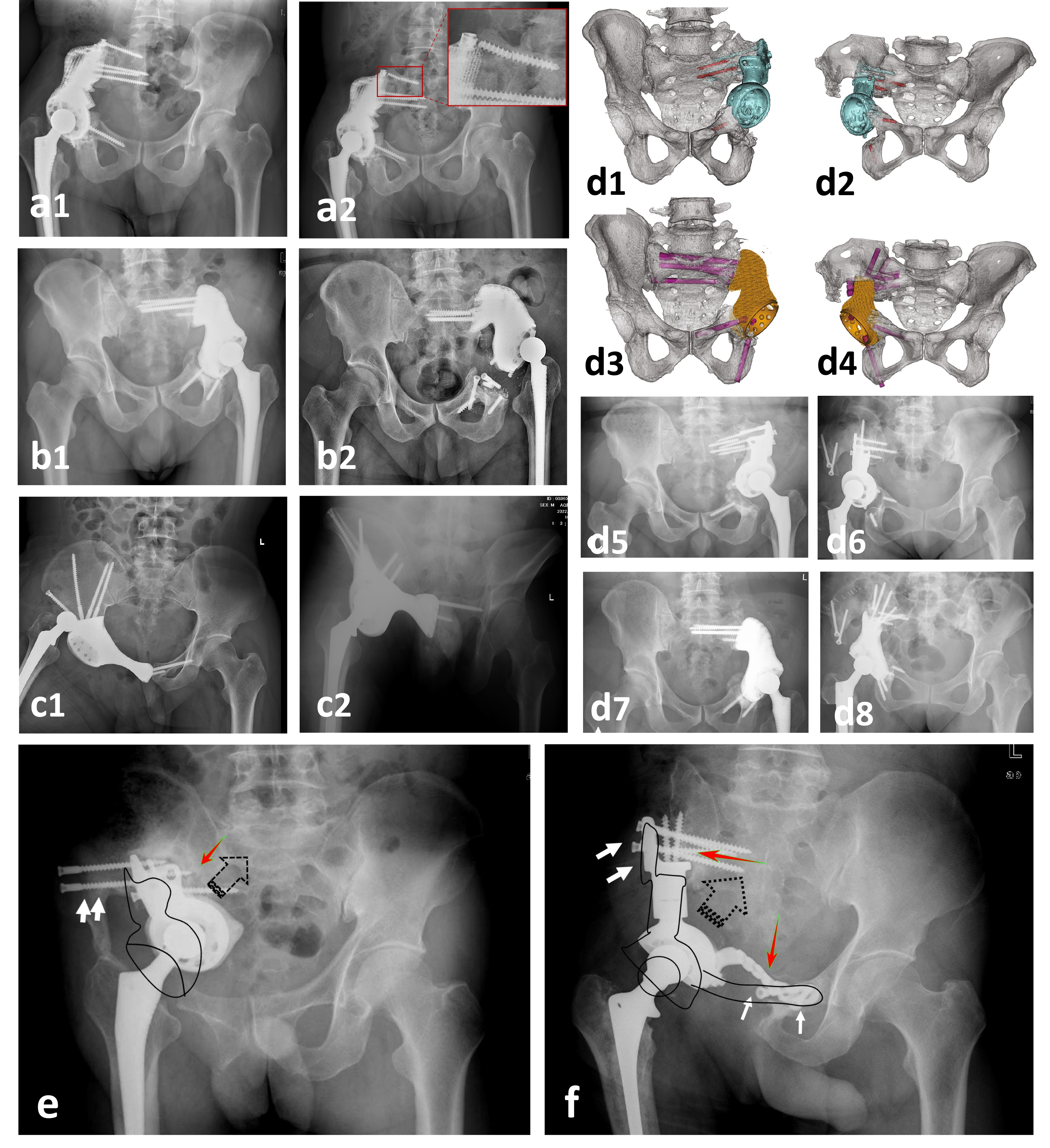

Supplement: Supplementary file 3 — Additional file 3. Fig. S3 Typical postoperative complications in 3D-printed prosthetic hip reconstruction surgery: a Screw Fracture: Day 2 Postoperative X-ray (a1). One Year Postoperative X-ray (a2) displays a screw fracture (a2) at the uppermost part of the sacroiliac joint, evident by the red mark. Notably, the patient remained asymptomatic, and conservative observation was chosen. b Aseptic loosening: Postoperative 1-year Pelvic X-ray (b1) reveals loosening and fracture of the ischial screw. Postoperative 2-year X-ray (b2) shows loosening at the prosthesis-bone interface and multiple screw failures. c Hip Dislocation: Three Days Postoperative X-ray (c1) reveals hip dislocation. Successful closed reduction under general anesthesia was performed (c2). d Design and Application of 3D-Printed Hemipelvic Endoprosthesis for Revision of Aseptic Loosening: Preoperative Simulation d1, d2 Depicted endoprosthesis migration and fractured screws. Illustrations d3, d4 Demonstrated design of 3D-printed custom hemipelvic endoprosthesis and screw fixation. Pre-revision Radiographs d5, d6 Displayed aseptic loosening, screw fracture, and endoprosthesis migration. Post-implantation Radiographs d7, d8 Revealed successful reconstruction with custom hemipelvic endoprosthesis. e Three Years Post-Surgery: Pelvic X-ray showed suboptimal integration at modular hemipelvic endoprosthesis interface and inward acetabular cup movement. f Five Years Post-Surgery: Pelvic X-ray indicated inadequate integration at modular hemipelvic endoprosthesis interface, with pubic plate deformation and bending. (Reprinted with permission from Ref [68] ©2021 BMC Surgery). [file 13018_2024_4697_MOESM3_ESM.png]
